# Contamination Issue in Viral Metagenomics: Problems, Solutions, and Clinical Perspectives

**DOI:** 10.3389/fmicb.2021.745076

**Published:** 2021-10-20

**Authors:** Henryk Jurasz, Tomasz Pawłowski, Karol Perlejewski

**Affiliations:** ^1^Department of Immunopathology of Infectious and Parasitic Diseases, Medical University of Warsaw, Warsaw, Poland; ^2^Division of Psychotherapy and Psychosomatic Medicine, Department of Psychiatry, Wrocław Medical University, Wrocław, Poland

**Keywords:** viral metagenomics, virome, contamination, low-biomass sample, virus

## Abstract

We describe the most common internal and external sources and types of contamination encountered in viral metagenomic studies and discuss their negative impact on sequencing results, particularly for low-biomass samples and clinical applications. We also propose some basic recommendations for reducing the background noise in viral shotgun metagenomic (SM) studies, which would limit the bias introduced by various classes of contaminants. Regardless of the specific viral SM protocol, contamination cannot be totally avoided; in particular, the issue of reagent contamination should always be addressed with high priority. There is an urgent need for the development and validation of standards for viral metagenomic studies especially if viral SM protocols will be more widely applied in diagnostics.

## Introduction

Next-generation sequencing (NGS) techniques combined with the development of computational tools led to an explosion of metagenomic studies in the past decade (Chiu and Miller, 2019; Lewandowski et al., 2019). Metagenomics is defined as direct analysis of the whole microbial communities based on DNA/RNA extracted from clinical or environmental samples (Huson and Mitra, 2012). Such analysis allows for the detection of known and unknown microorganisms and provides insights into the pathogen–host interactions, epidemiology, ecology, and evolution of organisms found across various ecosystems (Forbes et al., 2017; Chiu and Miller, 2019). Although microbial research remains dominated by bacterial 16S rRNA gene sequencing studies, new techniques were also used for viral analysis (Ladner et al., 2014; Moustafa et al., 2017; Kufner et al., 2019). Shotgun metagenomics (SM) is currently the most widely used technique to analyze viral DNA and RNA in a given environment (Conceicao-Neto et al., 2015; Forbes et al., 2017) and was successfully introduced into clinical practice to support diagnosis of systemic infections and occasionally identified a number of novel viral species (Palacios et al., 2008; Foulongne et al., 2011; Lipowski et al., 2017).

While SM is being used to characterize the virome using various workflows, it still faces numerous challenges, including the decision regarding best extraction and sequencing methods, the need for host genomic background depletion, the necessity of access to computational resources and highly specialized bioinformaticists, and providing relevant clinical data fast enough to be of clinical value (Schlaberg et al., 2017; Boers et al., 2019). Overall, SM approach has allowed for comprehensive surveys of never-before-seen viral communities (Moreno-Gallego et al., 2019; Waldvogel-Abramowski et al., 2019; Perlejewski et al., 2020b). However, SM also detects external contaminant nucleic acids and cross-contaminations, which can affect the interpretation of microbiome data (Xu et al., 2013; Laurence et al., 2014). So far, the issue of contamination in microbial sequencing studies was mostly discussed in regard to amplicon target sequencing (ATM); (16S rRNA gene sequencing) and was largely focused on bacterial bias (Karstens et al., 2019). Such contamination effects are common, as several studies have found contaminant microbial DNA in laboratory reagents and laboratory surfaces (Salter et al., 2014; Eisenhofer et al., 2019; Stinson et al., 2019). While several groups have also reported on the presence of genomic contaminants in viral SM data, there are no established criteria for examination and/or reporting of contamination in virome-focused studies (Moustafa et al., 2017; Zolfo et al., 2019; Perlejewski et al., 2020a). The current review emphasizes the impact of contaminants on viral studies, especially when using low-biomass samples, and proposes recommendations to minimize its effect.

## Sources of Contamination in Microbiome Studies

**FIGURE 1:**
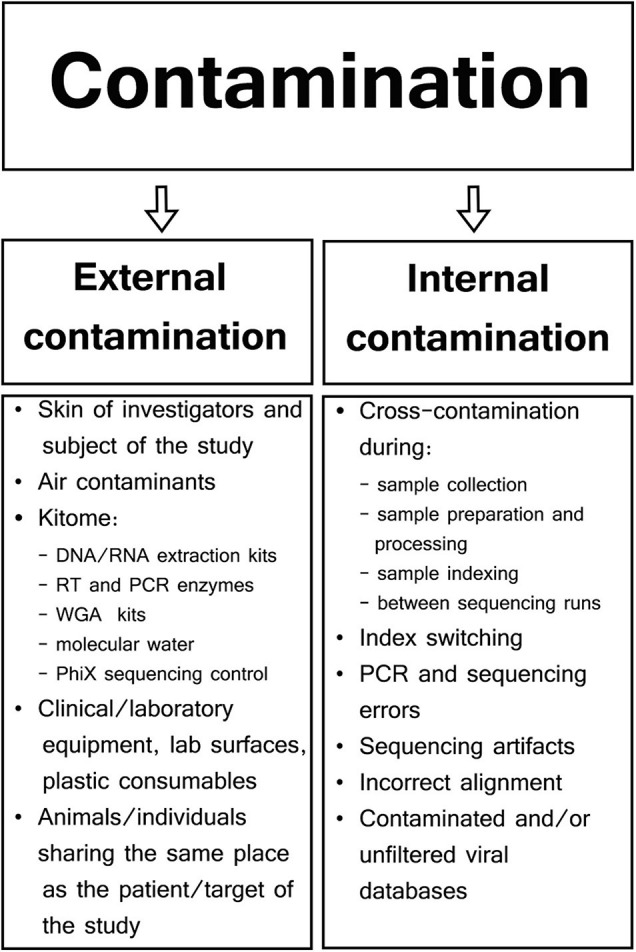
Types and sources of contamination in viral metagenomic studies.

Different types of samples and SM protocols affect the composition of genetic background found in viral metagenomics. Therefore, contaminants may be represented by external host/human or bacterial DNA, as well as sequencing reads aligned to genomes of a non-sample viral, fungal, protozoal, or even plant species (Perlejewski et al., 2015; Moustafa et al., 2017; Asplund et al., 2019). Specific contaminants are often not even reported in viral metagenomic studies as most viral SM research is focused only on viral hits, rarely aligning NGS reads to genomes other than host and viral. There are two major types of contaminants in viral SM studies: external or internal contamination (Figure 1; Davis et al., 2018; Eisenhofer et al., 2019).

### External Contamination

External contamination originates from the outside of samples during specimen collection and preparation and can include skin of patients or investigators (Kitchin et al., 1990; Meadow et al., 2015), clinical and laboratory equipment (Mukherjee et al., 2015; Llamas et al., 2017), collection tubes (Motley et al., 2014), contaminated laboratory surfaces or air (Bittinger et al., 2014), extraction kits, polymerase chain reaction (PCR) reagents (Grahn et al., 2003; Tilburg et al., 2010; Salter et al., 2014), or even molecular biology-grade water (Nogami et al., 1998; Kulakov et al., 2002; Keki et al., 2013). Manufacturers usually do not guarantee the absence of contaminating DNA in their products, and those reagents/kits that are sold as sterile may contain low-abundance external DNA (van der Horst et al., 2013). Generally, most external contaminations in microbiome studies have their own unique profile specific to particular reagents and kits; therefore, they are often referred to as kitome and are largely undistinguishable from microbiome signals derived from analyzed samples (van der Zee et al., 2002; Salter et al., 2014; Sabatier et al., 2020). Although a specific kitome can be detected and characterized, the types and quantities of reagent contaminants vary between different extraction/PCR kits and batches of the same reagent (Salter et al., 2014). True DNA/RNA signals are reproducible and associated with individual samples; however, reagent contamination signals are linked predominantly to specific batches or even reagents lots (Salter et al., 2014; de Goffau et al., 2018). For example, Glassing et al. (2016) analyzed MoBio DNA Extraction kit (QIAGEN; Hilden; Germany) and showed that 69% of dominating bacterial genera were the same in different lots of the kit, whereas the composition of minor genera was lot-dependent. Therefore, it has been recommended to process all samples in a particular project using the same batches/lots of reagents and to consider kit batches as a factor in the statistical analysis whenever multiple batches are used (Kim et al., 2017).

It seems that neither laboratories nor sequencing facilities are free from contamination, and this external DNA noise can change over time (Salter et al., 2014). For example, Weyrich et al. (2019) analyzed ultraclean ancient DNA laboratories for over 5 years and three modern molecular biology laboratories for 1 year and found that each one had its own unique microbial profile that changed over time according to the month and season. In another study, three different laboratories performed 16S rRNA sequencing of the same *Salmonella bongori* control using different batches of the same extraction kits (FastDNA Spin Kit For Soil; MP Biomedicals, Santa Ana, CA, United States) and obtained three different microbial profiles. This variation in the contaminant content could be the result of differences between kit batches and other reagents or may represent contaminants specific for each laboratory environment and investigators (Salter et al., 2014; Kim et al., 2017).

Extraction kits seem to be the major source of nucleic acids external noise in microbiome studies (Evans et al., 2003; Salter et al., 2014; Smuts et al., 2014; Zhi et al., 2014; Sabatier et al., 2020). Glassing et al. (2016) identified 88 bacterial genera in commonly used DNA extraction kits, and it was estimated that 10–50% of the bacterial profiles in lower-airway human samples are contaminants, and their main source are extraction kits (Drengenes et al., 2019). Commercial extraction kits were found to contain a higher microbial diversity and several more human-associated bacterial taxa when compared to in-house extraction protocols (Weyrich et al., 2019). A different genetic background with significant higher prevalence of contaminants was reported for manual compared to automated extraction systems (Sabatier et al., 2020). The latter is not unexpected as manual extractions require a higher number of manual transfer steps than single-tube spin-column approach, and thus, the risk of external contamination is increased.

RNA sequencing is more susceptible to contamination than DNA sequencing due to the presence of the extra reverse transcription (RT) step (Strong et al., 2014). In addition, it was found that commercially available RT enzymes can contain viral contaminants such as equine infectious anemia virus or murine leukemia virus (MuLV); (Zheng et al., 2011; Wally et al., 2019).

DNA and RNA sequencing SM protocols may include an amplification step to generate sufficient amount of DNA/cDNA for sequencing libraries (Malboeuf et al., 2013). A number of studies documented the presence of external DNA in various commercial polymerases (Bottger, 1990; Schmidt et al., 1991; Hughes et al., 1994); for example, microbial contaminants were reported in six commercially available *Taq* polymerases (Iulia et al., 2013). It was estimated that the amounts of contaminants in recombinant *Taq* polymerase range between 10 and 1,000 genome equivalents of microbial DNA per unit of enzyme (Spangler et al., 2009). Other potential sources of contaminants could also include PCR buffers or MgCl_2_ stocks, as well as primers prepared with water-containing contaminant DNA (Stinson et al., 2019). Considering the nature of SM and the necessity to analyze low-biomass samples, whole-genome amplification (WGA) is often used for the generation of templates suitable for sequencing (Thoendel et al., 2017). When three commercial WGA-DNA kits (Illustra V2 Genomiphi, Illustra single cell Genomiphi, and Qiagen REPLI-g single cell kits) were tested, it has been found that each contained a wide variety of microbial contaminant DNA (Thoendel et al., 2017). The origin of DNA background noise in WGA methods could come from amplification of contaminant DNA or from non-specific extension of random primers (Blainey and Quake, 2011). However, the consistent and highly specific contamination profile found in most individual WGA-DNA kits suggests the dominant role of the former (Thoendel et al., 2017). DNA background was reported in studies using WGA-RNA kits for the analysis of cerebrospinal fluid (CSF) and synovial fluid samples (Malboeuf et al., 2013; Perlejewski et al., 2015, 2016; Masters et al., 2018). WGA-RNA sequencing performed on clinical samples (CSF, swabs, and serum) and surrogate CSF samples (spiked with three 1:100 dilutions of influenza A H3N2 virus) using WTA2 kit (Sigma-Aldrich, St. Louis, MO, United States) resulted in the detection of a wide range of bacterial and viral contaminants. However, it should be noted that this background noise could have also originated from extraction kits and reagents used for the depletion of host genetic material (Oechslin et al., 2018).

The final step of wet-laboratory SM protocols is sequencing (Garmaeva et al., 2019). Currently, the most widely used method due to low costs, high yield, and wide availability is sequencing by synthesis marketed by Illumina (San Diego, CA, United States) (Kim et al., 2020). Despite numerous advantages, Illumina sequencing platforms share common challenge related to phage PhiX174 (approximately 5.3 kb) control used for quality and calibration assessment (Manley et al., 2016). While PhiX174 sequences should be removed from the final data, Mukherjee et al. (2015) reported that approximately 5.5% of publicly available microbial genomes in the Integrated Microbial Genomes database are contaminated by PhiX174, and 10% of them has been published in peer-reviewed scientific papers.

### Internal Contamination

Cross-contamination is the most challenging internal contamination source when compared among the other numerous sources of internal contamination encountered in microbial sequencing (Olomu et al., 2020). This form of contamination results from transfer of genetic material, amplicons, or barcodes between reaction tubes or wells (Carlsen et al., 2012; Poore et al., 2020). Sample cross-contamination can occur at different steps throughout the whole SM protocol because of incorrect pipetting, accidental splashes of liquids, generation of aerosols, incorrect tube opening, or plate cover removal (Tamariz et al., 2006; Joung et al., 2017). The risk of sample cross-contamination increases when a large batch of samples undergoes extraction and/or library preparation, especially when using tube strips without individual caps, or when using reaction plates (Lejal et al., 2020; Olomu et al., 2020). Specimen-to-specimen cross-contamination was found to be significantly more common in high-throughput whole-genome sequencing (HT-WGS) in comparison to Sanger sequencing when influenza A/H3N2 virus from nasal/nasopharyngeal/throat swabs was analyzed (Lee et al., 2016). Well-to-well contamination affects primarily neighboring samples, but occasionally even those 10 wells apart (Minich et al., 2019). In a study conducted by Minich et al. (2019) on no-template controls (NTCs), 47.5% of blanks for tubes and 95.7% of blanks for plate DNA extractions had evidence of well-to-well contamination. This contaminating effect was more common in samples with low biomass, thus negatively affecting microbial alpha and beta diversity metrics (Minich et al., 2019). To limit well-to-well contamination, it was proposed to keep a minimum of four-well gap between high- and low-biomass samples (Olomu et al., 2020).

Another type of cross-contamination is run-to-run contamination observed for MiSeq (Illumina, San Diego, CA, United States) sequencers, which may manifest itself for as many as seven sequential runs following the original run (Brumme and Poon, 2017; Eisenhofer et al., 2019). However, modifications to the post run wash procedure, mainly via the addition of a bleach wash, largely solved this problem (Brumme and Poon, 2017).

Another type of internal contamination occurs as a phenomenon called “index hopping” or “index switching” and is the main cause of incorrect sample assignment of sequencing reads in multiplexed pooled libraries (Griffiths et al., 2018). Index hopping refers to incorrect read assignment from a given NGS library based on assignment to a barcode belonging to a different one sequenced in the same pool (Costello et al., 2018). This effect is largely due to an excess of free index primers, which, together with the cluster generation reagents, randomly ligate to other samples pooled together in the sequencing run (Carlsen et al., 2012; Sinha et al., 2017; Costello et al., 2018). According to Sinha et al. (2017) in a multiplexed pool of samples sequenced on Illumina platform HiSeq 4000, up to 5–10% of all sequencing reads are misassigned from one sample to another. Index hopping is also a well-known phenomenon reported for the MinION (Oxford Nanopore Technologies, Oxford, Great Britain) sequencer where 0.056% of reads were found to have incorrectly assigned barcodes (Xu et al., 2018). Index switching reduces the value of negative controls in sequencing runs as NTCs and analyzed samples may contain the same sequences; thus, true signals cannot be distinguished from background noise (Hornung et al., 2019). To reduce index switching, unique dual-indexing and dual-matched indexed adapters with unique molecular indices are recommended (MacConaill et al., 2018; van der Valk et al., 2020).

Internal contamination in microbiome sequencing could also be caused by DNA damage and polymerase errors (Brandariz-Fontes et al., 2015; Hornung et al., 2019). In one study evaluating 13 commercial polymerases, it was found that enzyme choice has a large impact on the proportion of correct reads recovered from multiple gene sequencing run (from 17 to 71%) (Brandariz-Fontes et al., 2015). Nucleotide misincorporation, generation of chimeric sequences, or variation in efficiency of amplification of high and low GC fragments can arise from amplification bias (Brodin et al., 2013; Shugay et al., 2014). Sequencing of GC-poor regions on Illumina platforms is typically less efficient, which limits uniform read coverage across the genome, thus affecting viral genome assembly in SM analysis (Kozarewa et al., 2009; Chen Y. C. et al., 2013). A partial solution for amplification errors is offered by the use of high-fidelity polymerases, which are characterized by up to 100 times lower error rates and lower chimera generation rates (Sze and Schloss, 2019). Importantly, PCR conditions also play a significant role in generation of internal contaminants, and it has been demonstrated that a lower number of PCR cycles results in a lower signal-to-noise ratio in microbial profiling studies (Quail et al., 2011; Sze and Schloss, 2019). Errors can also arise during various parts of sequencing procedure (cluster amplification, sequencing cycles, image analysis), resulting in error base calling of approximately 0.1–1%, depending on sequencing platform (Fox et al., 2014).

Finally, internal contamination may be the result of erroneous bioinformatics reads classification (Hornung et al., 2019; Ye et al., 2019). In the current literature, there are dozens of virus-specific classification workflows that are based on different strategies using anything from simple one-step tools to analyses that combine five or more steps and a variety of algorithms for virome analysis (Wommack et al., 2012; Zhao et al., 2017; Nooij et al., 2018; Kieft et al., 2020). Nooij et al. (2018) evaluated 49 different workflows suitable for viral classification and found that the positive predictive value was generally high (>75%), although some classifiers had lower and varied precision scores: IMSA+A (9%), Kraken (34%), NBC (49%), and vFam (3–73%). Taxonomic classifiers are also associated with different default thresholds for false-positive signal detection (from 0.01 to 0.5%), which results in tens (Bracken, MetaPhlAn2) or even thousands (Centrifuge, CLARK, Kaiju, MMseqs2, PathSeq) of false-positive hits, depending on the workflow (Ye et al., 2019).

False reads assignments in microbiome studies may also arise from contamination of publicly available databases. For instance, more than 330,000 bp in the reference genomes of *Plasmodium gaboni* and *Plasmodium falciparum* were found to be contaminated with human genome (Kryukov and Imanishi, 2016). Merchant et al. (2014) discovered that *Neisseria gonorrhoeae* TCDC-NG08107 genome submitted to GenBank contained fragments of cattle and sheep genomes. Similarly, The Cancer Genome Atlas database was found to be contaminated by human papillomavirus type 38 originating from RNA sequencing of human endometrial samples (Kazemian et al., 2015). The previously mentioned contamination with PhiX174 illustrates the scale and range of microbial database contamination (Mukherjee et al., 2015).

## Contamination in Low-Biomass Samples

The impact of contamination is especially significant for low microbial biomass samples where the proportion of background noise increases with the decrease of input template (Malboeuf et al., 2013; Karstens et al., 2019). The quantity of biomass can be evaluated by comparing the amount of extracted DNA/RNA from the studied sample to the volume of genetic material isolated from NTCs in the same SM protocol. Samples specified as low biomass typically contain the amount of DNA/RNA similar to NTCs, whereas rich samples contain significantly more genetic material than blank controls (Lauder et al., 2016). Contaminants can easily dominate in low-biomass samples generating background noise that is much higher than true signal originating from the target virus (Malboeuf et al., 2013; Salter et al., 2014). So far, a wide variety of environmental and clinical samples containing low viral biomasses have been studied with SM workflows including air (Prussin et al., 2019), glacier ice (Zhong et al., 2020), human skin (Tirosh et al., 2018), nasal swabs (Altan et al., 2019), and CSF (Perlejewski et al., 2020b; Perlejewski et al., 2020c). Most widely used library preparation kits for sequencing require inputs as low as 1 ng of DNA (e.g., llumina Nextra XT), but this may still be unattainable for some low-biomass samples. Based on our own experience and other published studies, some biological samples such as CSF yield <1 ng of DNA/RNA after typical 200 μL extraction, and viral load found in this material is often in the range of 100 copies/mL (Poissy et al., 2012; Bradshaw and Venkatesan, 2016; Perlejewski et al., 2020b). According to estimations by Garmaeva et al. (2019) 1 g of stool yields between 0.22 and 0.87 ng/μL of bacteriophage DNA (when using 50–200 μL of elution volume) signaling the need for application of more robust extraction and amplification methods, generating up to picograms of DNA (Garmaeva et al., 2019). To generate sufficient amount of material for library preparation and sequencing, new products based on techniques, such as multiple displacement amplification (Spits et al., 2006), linker amplification shotgun libraries (Bittinger et al., 2014), sequence-independent single-primer amplification (Chrzastek et al., 2017), or single-primer isothermal linear amplification (Ribo-SPIA), were introduced (Dafforn et al., 2004). Commercial kits such as NuGEN’s Ovation RNA-Seq System V2, which is based on Ribo-SPIA, can generate sufficient amount of cDNA for library preparation from as little as 500 pg of RNA with sufficient coverage and read count when sequencing as little as 100 copies of HIV RNA (Malboeuf et al., 2013). Although all these methods solve the problem of insufficient material for sequencing in low-biomass samples, they neither reduce nor distinguish contaminants from true signals. Moreover, as previously mentioned, there is some evidence that these kits can be the source of extra genetic background themselves (Thoendel et al., 2017; Oechslin et al., 2018; Perlejewski et al., 2020a).

Another common problem specific for ultralow-biomass samples (input < 50 pg) is the high level of read duplication reaching 70%, whereas it was reported to be only 0.5–2% with high DNA input samples (>50 ng) (Garmaeva et al., 2019). This may generate a significant bias in quantitative analysis when different communities are compared to each other, and more irreproducible background noise is being amplified with decreasing sample biomass (Salter et al., 2014; Garmaeva et al., 2019; Erb-Downward et al., 2020). Finally, low-biomass samples require extra steps during extraction and library preparation, which increase the likelihood of external and internal contamination (Salter et al., 2014; Rawlinson et al., 2019).

## Contaminants Detected in Viral Studies

### Viral Contaminants

Viral contaminants seem to be highly relevant among all types of contaminants encountered in viral metagenomic research, and they can, occasionally, significantly impact results interpretation, as was the case in the study by Xu et al. (2013) who identified National Institutes of Health–Chongqing virus (NIH-CQV) in patients with seronegative hepatitis. Although this particular pathogen was detected in 70% of hepatitis patients and in 0% of 45 healthy controls, it was later determined that this novel hybrid parvovirus-like virus was a contaminant from silica column–based RNA extraction kit (QIAamp MinElute Virus Kit; Qiagen, Hilden, Germany) (Smuts et al., 2014). The observed lack of NIH-CQV presence in healthy control subjects was probably related to lot-to-lot differences in the degree of spin column contamination (Naccache et al., 2014b). A year later, *Acanthocystis turfacea* chlorella virus 1 (ATCV-1) was proposed to be linked with the cognitive decline in humans after it was found in oropharyngeal samples collected from adults without current and past psychiatric disorders within a study that included measures of cognitive functioning (Yolken et al., 2014). ATCV-1 is of algal origin and was later found to be a part of kitome of commercial DNase and RNA extraction kits (Kjartansdottir et al., 2015). In general, most of reported contaminants in viral metagenomic studies seem to come from DNA and RNA extraction kits (Asplund et al., 2019).

In another study, a silica column–based kit (QIAamp viral RNA mini kit; Germany) was found to generate background noise of *Iridoviridae, Circoviridae, Baculoviridae*, and *Genomoviridae* sequences (Ngoi et al., 2016). In a recent study comparison of three extraction kits for metagenomic analysis of respiratory viruses, 19, 28, and 55 viral families were identified in NTCs using eMAG (bioMérieux, Marcy-l’Étoile, France), MagNA Pure 24 (Roche, Basel, Switzerland), and QIAamp Viral RNA Mini Kit extraction (Qiagen), respectively. Once again, the highest genetic background was found for the Qiagen kit, and it was composed of hits classified as *Siphoviridae, Myoviridae, Microviridae*, and *Podoviridae* (Sabatier et al., 2020). Various other reagents were also found to be a potential source of contamination, for example, BVDV−3 (bovine viral diarrhea virus 3) is a common contaminant in fetal bovine serum (Bergner et al., 2019), whereas MuLV is present in 17 human cell lines (Cao et al., 2015; Uphoff et al., 2015), as well as in reverse transcriptase–PCR reagents (Zheng et al., 2011; L’Huillier et al., 2019).

Separation of true signals from background noise is both extremely important and yet challenging in clinical settings. Bacteriophages are particularly common among a variety of contaminants encountered in clinical metagenomics (Naccache et al., 2014a; Ngoi et al., 2016; Asplund et al., 2019; Sangiovanni et al., 2019) and may disrupt the quantitative picture of virome, whereas sequences of eukaryotic viruses may be falsely associated with diseases (Xu et al., 2013). That was the case in the study linking febrile Kenyan adults with Kadiprio virus, which was initially considered to be the causative agent but was eventually found to be a part of QIAamp Viral RNA Mini Kit (Qiagen) kitome (Ngoi et al., 2016). In a recent study, Mollerup et al. (2019) used NGS to search for viruses in human cancers and found Merkel cell polyomavirus (MCPyV) in Merkel cell carcinomas. However, close similarity of all MCPyV sequences found across samples allowed studies to conclude laboratory surfaces as the source of contamination (Foulongne et al., 2011; Mollerup et al., 2019). In our previous viral SM studies, we often found pandoravirus sequences in CSF of patients with encephalitis and in NTCs (Perlejewski et al., 2015; Bukowska-Osko et al., 2016; Moustafa et al., 2017). After closer analysis of these sequences (low-complexity reads with nucleotide tandem repeats), they were determined not to represent true signals, but sequencing artifacts and/or contaminants originating in laboratory reagents (Hjelmso et al., 2017; Waldvogel-Abramowski et al., 2019).

So far, there are very few studies addressing the issue of viral contamination in viral sequencing (Naccache et al., 2014b; Moustafa et al., 2017; Asplund et al., 2019). The most comprehensive was the one by Asplund et al. (2019) that evaluated 712 sequencing libraries prepared using several different protocols and found almost 500 viral hits associated with laboratory components. Similar to our observations, more contaminants were present in RNA sequencing protocols than those using DNA as a starting material. Most viruses reported by Asplund et al. (2019) were bacteriophages (60%), which is also consistent with our own studies in which phages constituted 96 and 77% of all viral sequences in CSF from encephalitis patients using RNA-based and DNA-based SM workflows, respectively (Perlejewski et al., 2020b). Viruses of non-human vertebrae hosts constituted approximately 12% of all viral contaminants (Asplund et al., 2019).

A frequent problem in viral SM studies is cross-contamination occurring when high viral-titer samples are simultaneously sequenced with low-biomass samples in the same sequencing lane (Moustafa et al., 2017). This is especially relevant when viral SM is performed using clinical samples, and overexpressed viral hits from one sample affect the viromes of other specimens. High-titer samples commonly contaminate low-biomass samples in the same sequencing run, and the rate of cross-contamination on Illumina platforms was reported to be approximately 0.05% (Deng et al., 2020). In nanopore sequencing, cross-contamination occurs when low- and high-titer samples are pooled; to remedy these problems, it was proposed to batch samples together according to viral loads (Lewandowski et al., 2019).

### Bacterial Contaminants

Bacterial contaminants affect both viral SM and ATM studies in a similar manner because of the same external origin of bacterial sequences, which are usually present in the kitome (Salter et al., 2014). In approximately 72% of virome samples, bacterial DNA is considered to be the most abundant contaminant. Surprisingly, a significantly higher bacterial background noise is present in virus enriched than in non-enriched metagenomic samples (Zolfo et al., 2019). These findings indicate that many virus-like particles (VLP)–targeting SM workflows fail in efficient virus enrichment and experience large contamination problems.

The predominant bacterial genera found in negative controls in ATM and SM studies are *Propionibacterium*, *Flavobacterium*, *Streptococcus*, *Burkholderia*, *Methylobacterium*, *Curvibacter*, *Ralstonia*, *Escherichia*, *Acinetobacter*, and *Stenotrophomonas* (Lauder et al., 2016; Weyrich et al., 2019). Salter et al. (2014) reported the presence of *Proteobacteria*, *Actinobacteria*, *Firmicutes*, *Bacteroidetes*, *Deinococcus-Thermus*, and *Acidobacteria* in blank controls in PCR-based 16S rRNA gene and SM studies. In a study using HT-WGS in six different sequencing centers, *Bradyrhizobium* was reported to be the most common bacterial contaminant genus (Laurence et al., 2014). Moreno-Gallego et al. (2019) found that more than 1% of bacterial reads identified in a fecal virome represented contamination and they belonged largely to *Firmicutes* phylum. This is compatible with the findings of Zolfo et al., who analyzed bacterial contaminants using measurements of bacterial small subunit ribosomal RNA gene (SSU rRNA). In 37 virome studies (analyzed environmental and human samples), SSU rRNA median ranged from 0 to 14.3% (approximately 1.2% per data set); (Zolfo et al., 2019).

### Host/Human Contaminants

In HT-WGS studies of such clinical samples as stool or CSF, host genomic reads are an integral part of whole metagenomes (Nakamura et al., 2009; Perlejewski et al., 2020c). Some investigators name all host reads as contaminants, as these sequences mask true signals and reduce assay sensitivity for pathogen detection (Malboeuf et al., 2013; Charre et al., 2020; Heravi et al., 2020). Moreover, overrepresentation of host sequences in large NGS data sets can extend the process of data analysis and require high and costly computational powers (Hasan et al., 2016).

The majority of human/host reads in WGS studies derived from the actual sample constitute a part of true genetic background; however, they reduce the sensitivity and sequencing coverage in microbial sequencing studies, especially for low-biomass samples (Chiu and Miller, 2019; Pereira-Marques et al., 2019). Clinical SM studies revealed that in such human-derived samples as nasopharyngeal aspirate, serum, and brain tissue, up to ∼95–99% of raw NGS reads derive from human DNA (Yang et al., 2011; Lipowski et al., 2017). Consequently, without a significant host genomic depletion, viral genome coverage is likely to be low even when high viral loads are present (Luk et al., 2015). In clinical settings, the minimum viral–host read ratio needed for viral identification is highly variable and species/sample/workflow-dependent. For instance, viral/human mRNA ratio of 0.0005% led to the discovery of MCPyV (Feng et al., 2008), whereas viral/human RNA ratio was 0.0135% when a new arenavirus causing febrile illness was first identified in patients who received solid organ transplants from a single donor (Palacios et al., 2008). In low-biomass clinical samples, human DNA/RNA overwhelms viral signals, but a variety of host depletion methods can partially remedy the problem by decreasing the background noise up to 3,100-fold with negligible loss of target virus (Oechslin et al., 2018). Unfortunately, with the reduction of host genomic contamination, an increase of non-host contaminants is common, especially when kitome-related signals are being amplified (Salter et al., 2014; Oechslin et al., 2018). Finally, some VLP purification methods such as CsCl density gradient ultracentrifugation efficiently remove host-derived DNA, but at the same time discriminate against particular viruses, thus affecting quantitative virome measurements (Kleiner et al., 2015).

### Other Contaminants

Bacterial and host-derived sequences are rarely reported in SM viral studies because NGS reads are often not aligned to comprehensive databases that include non-viral genomes. In SM studies on human nasopharyngeal samples and CSF, reads were mapping to plant, parasitic, fungal genomes, and even synthetic constructs (Nakamura et al., 2009; Perlejewski et al., 2015). These hits could have derived from various sources including reagents, sequencing errors, and erroneous classification, especially when using unfiltered and biased genome databases for alignment.

## Criteria for Virus Identification and Sequence Decontamination

In virus-targeted SM studies, it is critical to make an accurate distinction between true viral signals and contaminants (Xu et al., 2018; Asplund et al., 2019). This is especially difficult when low-biomass samples containing low viral loads are being analyzed (Malboeuf et al., 2013; Perlejewski et al., 2016). So far, a variety of SM workflows have been used for various samples using numerous wet-laboratory procedures and bioinformatics analysis, but a universally efficient approach is still unclear (Nakamura et al., 2009; Conceicao-Neto et al., 2015; Lewandowski et al., 2019).

SM viral protocols require validation and standardization before they can be used for routine clinical application (van Boheemen et al., 2020). The protocols used are highly dependent on the type of sample. For instance, stool and tissue samples are treated differently (homogenization, filtration, DNA/RNA extraction, or nuclease treatment) than low-biomass samples such as CSF, human skin, or nasal swabs (e.g., required preamplification steps) (Hall et al., 2014; Sabatier et al., 2020). Thus, any future standardized SM clinical viral protocols must take into consideration sample type and the expected viral pathogen (either DNA or RNA-based approach) (Schlaberg et al., 2017; Kufner et al., 2019). Moreover, the same factors may affect the decision on sequencing parameters such as sequencing depth, which specifies how many times each base in a genome should be covered by NGS reads (Deng et al., 2020). This parameter is associated with the abundance of target virus, which affects the sensitivity of applied workflows (Malboeuf et al., 2013; Pereira-Marques et al., 2019). Another factor to consider is sequencing breadth, which specifies what portion of a genome should be sequenced for a reliable identification (Wylie et al., 2018). Ladner et al. (2014) proposed five categories to define different genome standards in viral-targeted sequencing beginning with a “standard draft,” representing a low coverage with at least 50% of a draft genome candidate recovered (frequent for low-biomass samples with low viral loads). On the opposite site, a “finished” category requires high coverage rates (400–1,000×) and represents cases when a complete viral consensus genome sequence is obtained, combined with complete population-level characterization of genomic diversity (Ladner et al., 2014).

So far, there are no universal criteria for positive virus species identification in HTS-WGS analyses. Currently, it seems that the gold standard for microbial confirmation after identification by metagenomics is PCR or Sanger sequencing (Yu et al., 2016; Fang et al., 2018; Wylie et al., 2018; Holmes, 2019). Theoretically, even a one virus-specific NGS read in SM could indicate a true signal. In the already mentioned study, a novel arenavirus was identified in organ transplant setting after only 14 virus-specific sequences were detected by SM (Palacios et al., 2008). Liu et al. (2020) proposed that a positively identified viral taxon should be represented by at least two unique sequencing reads detected by the same or a different technique, whereas detection of reads mapping to at least three non-overlapping genome regions was required to identify virus in CSF in the studies conducted by Schlaberg et al. (2017) or Miller et al. (2019). Reads dispersed across the whole genome and with high coverage indicate the presence of true viral signals, but isolated and/or repeated viral sequences found across samples from the same run suggest sequencing artifacts (Asplund et al., 2019). In a study evaluating viral SM workflow in a tertiary diagnostic unit, positive viral identification required detection of at least three viral reads distributed across the whole genome with a high coverage score. Furthermore, the number of reads for the target virus had to be at least 100 times higher than in negative controls and other samples (Kufner et al., 2019). This approach is balanced as it takes into account the high possibility of cross-contamination between samples and NTCs, whereas many microbiome studies disqualified all sequences found in negative controls (Dunn et al., 2013; Karstens et al., 2019). A blacklist method assembles a catalog of specific contaminants found in NTCs in a given study and/or sequencing center and uses them in an algorithm to exclude matching sequences from WGS data sets (Ye et al., 2019). However, it is well-documented that true signals can also occur in NTCs as part of the index switching phenomenon (Callahan et al., 2017; Sinha et al., 2017; Costello et al., 2018; Larsson et al., 2018). It was shown that index switching ratios are higher in NTCs than in template-containing samples, indicating that at least several NTCs should be included in each sequencing run (Asplund et al., 2019). This approach allows for the detection of even sporadic contaminants, which is relevant if the decontamination is based on removal of sequences below a specified read/species abundance threshold (Lazarevic et al., 2016; Asplund et al., 2019).

Different thresholds were used in SM viral studies to distinguish between true and false-positive hits; for example, Guerin et al. (2020) proposed a threshold of >100 hits. In a study by Wylie et al. (2018) using pools of clinical samples (CSF, blood, plasma urine, swabs), the threshold of 0.1% of total reads for each virus expected in the appropriate sequencing pool was applied to limit the impact of index switching. In another study using VLP enrichment protocols, a relative read count threshold of 0.01% was set based on an empirical index contamination rate (O’Flaherty et al., 2018).

Viral identification is currently supported by numerous computational algorithms and open-source programs, such as VirSorter (Roux et al., 2015), VirusFinder (Wang et al., 2013), VirusSeeker (Zhao et al., 2017), VirusSeq (Chen Y. et al., 2013), VirusDetect (Zheng et al., 2017), and ViromeScan (Rampelli and Turroni, 2018). Some of the algorithms/pipelines [ViralFusionSeq (Li et al., 2013), Virana (Schelhorn et al., 2013), VERSE (Wang et al., 2015)] even allow for the detection of viruses integrated into the host genomes. Another group of useful programs such as MARVEL (Amgarten et al., 2018), PhagePhisher (Hatzopoulos et al., 2016), or Phage_Finder (Fouts, 2006) are designed to detect phages in metagenomic data sets. Special caution is required when interpreting the results of viral mining software applied in mixed metagenomes as they contain more computationally derived internal contamination compared to virus-specific data sets. Zolfo et al. (2019) showed that assembly carried out in poorly enriched metagenomes increases the number of contigs falsely classified as viral. More than 20% of assembled reads were assigned as viral in approximately 12% of metagenomic poorly enriched samples. This indicates a significant presence of viral false-positives found in data sets containing high representation of bacterial genomes (Zolfo et al., 2019).

Contamination in metagenomic studies can also be reduced or even removed using open-source software, such as R package decontam, which takes advantage of two observations: (i) contaminants are found at higher frequencies in low-titer samples, and (ii) their presence is more common in negative controls than in true samples (Davis et al., 2018). A similar application presents DecontaMiner, which uses a subtraction approach to detect contaminations by bacteria, fungi, and viruses from different sources (Sangiovanni et al., 2019). A much more virome-focused software is ViromeQC, which is designed for benchmarking and quantifying non-viral contamination in VLP-enriched projects. It uses three microbial markers: SSU-rRNA, large subunit rRNA gene, and 31 prokaryotic single-copy markers. In addition, ViromeQC calculates viral enrichment score measuring the quality of VLP enrichment protocol (Zolfo et al., 2019). Finally, R packages such as microDecon (McKnight et al., 2019) or CroCo (Simion et al., 2018) are designed to efficiently and correctly detect cases of cross-contamination in studies using metabarcoding.

## Concluding Remarks

Evolution of NGS and WGA methods has allowed for the development of numerous metagenomic workflows, which were successfully applied in viral-focused studies across various environments (Conceicao-Neto et al., 2015; Kohl et al., 2015; Perlejewski et al., 2020b). Regardless of the specific viral SM protocol, contamination cannot be totally avoided, and in particular, the issue of reagent contamination should always be addressed with high priority (Asplund et al., 2019). So far, the problem of contamination was mostly studied in 16S rRNA profiling, and only a few viral SM studies used NTCs or reported kitome sequences characteristic for their protocols (Grahn et al., 2003; Karstens et al., 2019).

In the present article, we described the most common sources and types of contamination found in viral metagenomic studies, and we propose some basic recommendations for reducing the background noise (Table 1). There is an urgent need for the development and validation of standards in viral metagenomics, which would limit contamination bias, increase the quality of research, and allow viral SM protocols to be more widely applied in diagnostics.

**TABLE 1:** Recommendation for reducing contamination in viral metagenomic studies.

	**Recommendations**
General practices	• Use sterile laboratory equipment: tubes, tips with filter, decontaminated racks, and machines • Wear disposable protective coats, gloves, and face masks • Always decontaminate working area • Perform wet-laboratory work under laminar flow hood • Perform all steps in dedicated laboratory areas: create separate preamplification, amplification, and postamplification sites • Minimize the number of investigators in a project and record which samples were handled by a given technician
Sampling	• Avoid cross-contamination during sample preparation • Be aware that caging multiple laboratory animals in the same space may influence their microbial composition • Collect samples in sterile tubes • Avoid contamination derived from the skin or breath of the investigator • Use rich-biomass samples Maximize the sample volume for extraction when using low-biomass material
Reagents and wet-laboratory procedures	• Use the same types of reagents during the whole project Record all batches and lot numbers of all reagents used in a project • Minimize the number of steps in wet-laboratory workflow • Use dedicated extraction kits for low-biomass samples with low elution volumes • Keep in mind that silica column–based nucleic acid extraction kits are associated with numerous contaminants • Use highly purified enzymes and polymerases with high fidelity • Minimize the number of PCR cycles during amplification • Avoid using multichannel pipettes, sample plates, and strips without separate caps • If necessary make gaps in plates between samples • Use VLP enrichment workflows • Analyze the same biological samples in repeats
Sequencing	• Sequence all samples in a given project in the same sequencing center • Use unique dual barcoding • Sequence samples with similar viral titters in the same run • Minimize the number of PCR cycles during indexing
Controls	• Use blank and negative controls during sample preparation and extraction • Use non-template controls if amplification step is included • Use a variety of positive-control titrations to verify the accuracy of metagenomic workflow
Data analysis	• Create a list of contaminants specific for your viral metagenomic workflow and laboratory • Set your own threshold for contamination detection based on your results and experience • Align NGS reads to host and bacterial genomes to examine potential contamination • Set criteria for viral detection that include matching different regions of the viral genome with sufficient genome coverage • Align contigs rather than single NGS reads to viral genomes • Check the complexity of identified viral sequences to distinguish true signals from artifacts • Take into consideration sequencing error • Use verified and filtered viral databases for viral classification • Remove PhiX phage sequences before data upload • Use open-source decontamination software • Use dedicated software for viral detection and phage identification
Data interpretation and good practices	• For clinical diagnostic application, verify all potentially causative viral agents found in SM studies using PCRs • Pay close attention and be critical with regards to non-vertebrae viruses found in virome of vertebrae hosts • Perform batch/study/investigator associations with contaminants found in your data

## Author Contributions

KP, HJ, and TP: writing—original draft preparation and visualization. KP and HJ: conceptualization, data curation, and writing—review and editing. All authors contributed to the article and approved the submitted version.

## Conflict of Interest

The authors declare that the research was conducted in the absence of any commercial or financial relationships that could be construed as a potential conflict of interest.

## Publisher’s Note

All claims expressed in this article are solely those of the authors and do not necessarily represent those of their affiliated organizations, or those of the publisher, the editors and the reviewers. Any product that may be evaluated in this article, or claim that may be made by its manufacturer, is not guaranteed or endorsed by the publisher.
